# Overexpression of stearoyl-CoA desaturase 1 in bone marrow mesenchymal stem cells enhance the expression of induced endothelial cells

**DOI:** 10.1186/1476-511X-13-53

**Published:** 2014-03-20

**Authors:** Yuanshan Lu, Zihui Zhou, Jie Tao, Bang Dou, Mingjie Gao, Yue Liu

**Affiliations:** 1 Department of Transfusion, Shanghai First People’s Hospital, School of Medicine, Shanghai Jiao Tong University, 100, Haining Road, Shanghai 200080, China; 2 Department of Orthopedics, Shanghai First People’s Hospital, School of Medicine, Shanghai Jiao Tong University, 100, Haining Road, Shanghai 200080, China; 3 Department of Orthopedics, Shanghai First People’s Hospital Songjiang Branch, School of Medicine, Shanghai Jiao Tong University, 746, Zhongshan Middle Road, Shanghai 201600, China

**Keywords:** Stearoyl-CoA desaturase 1, Bone marrow mesenchymal stem cells, Endothelial

## Abstract

**Background:**

Bone marrow mesenchymal stem cells (BM-MSCs) are capable of differentiating into endothelial cells in vitro and acquire major characteristics of mature endothelial-like expression of vWF and CD31. SFAs and lipid oxidation products have been linked with postprandial endothelial dysfunction. Consumption of SFAs impairs arterial endothelial function, while a Mediterranean-type MUFA-diet has a beneficial effect on endothelial function by producing a decrease in levels of vWF, TFPI and PAI-1. Stearoyl-CoA desaturase 1 (SCD1), which converts SFA to MUFA, is involved in the cellular biosynthesis of MUFAs from SFA substrates. High expression of SCD1 is corresponded with low rates of fatty acid oxidation, therefore it might reduce inflammatory responses and be beneficial for the growth of induced endothelial cells. Overexpression of SCD1 in BM-MSCs might increase the growth of induced endothelial cells. The goal of this research is to study the relationship between overexpression of SCD1 and the expression of induced endothelial cells in BM-MSCs in vitro.

**Methods:**

The gene SCD1 was integrated into a lentiviral vector, and then 293 T cells were transfected by the connected product to produce a packaged virus. BM-MSCs were infected by the packaged virus. Cell culture and endothelial induction were performed. Fluorescent quantitative PCR of CD31, vWF and VE-cad was performed after 1 week and 2 weeks to test the growth of induced endothelial cells.

**Results:**

The mRNA amount of CD31, vWF and VE-cad of the SCD1 overexpressed group was statistically higher than that of the empty vector (EV) group and that of the normal group after 1 week and 2 weeks, respectively (p < 0.05). Immunocytochemical staining of CD31 or vWF was detected by visualizing red color.

**Conclusions:**

This study suggested that overexpression of SCD1 in BM-MSCs could increase the expression of induced endothelial cells in vitro.

## Background

Stearoyl-CoA desaturase 1 (SCD1), known as a ∆9 desaturase, is an integral membrane protein anchored in the endoplasmic reticulum [1]. SCD1 converts saturated fatty acids (SFAs) to monounsaturated fatty acids (MUFAs), which make up the primary storage of unsaturated fatty acids in human adipose tissue [2].

An inverse relationship between endothelial function and SFA has been shown in healthy adults [3]. Plasma SFA, which partly reflects dietary fat quality, has an important impact on endothelial function. Consumption of SFAs reduces the anti-inflammatory properties of HDL and impairs endothelial function [4]. In healthy adolescents, impaired endothelial function is significantly associated with high level of soluble ICAM-1, HOMA-IR and SFA [5].

Mesenchymal stem cells (MSCs) are multipotent stem cells that can be isolated from many human adult or fetal tissues. MSCs were found to differentiate into cartilage, bone, fat, muscle, and other connective tissues [6,7]. In endothelial differentiation system, human bone marrow-derived (CD105+, CD29+, CD34–) MSCs are capable of differentiating into endothelial cells in vitro and acquire major characteristics of mature endothelial-like expression of von Willebrand factor (vWF) and CD31 [8]. This will make bone marrow mesenchymal stem cells (BM-MSCs) attractive candidates for the development of autologous tissue graft. Overexpression of SCD1 in BM-MSCs might have advantages for the expression of induced endothelial cells by reducing the the amount of SFAs in human bodies.

Up to now, there has been no report in the literature of the relationship between the overexpression of SCD1 and the expression of induced endothelial cells in BM-MSCs. The goal of this research is to study the relationship between the overexpression of SCD1 and the expression of induced endothelial cells in BM-MSCs in vitro.

## Results

### Vector construction and virus packaging

**Figure 1:**
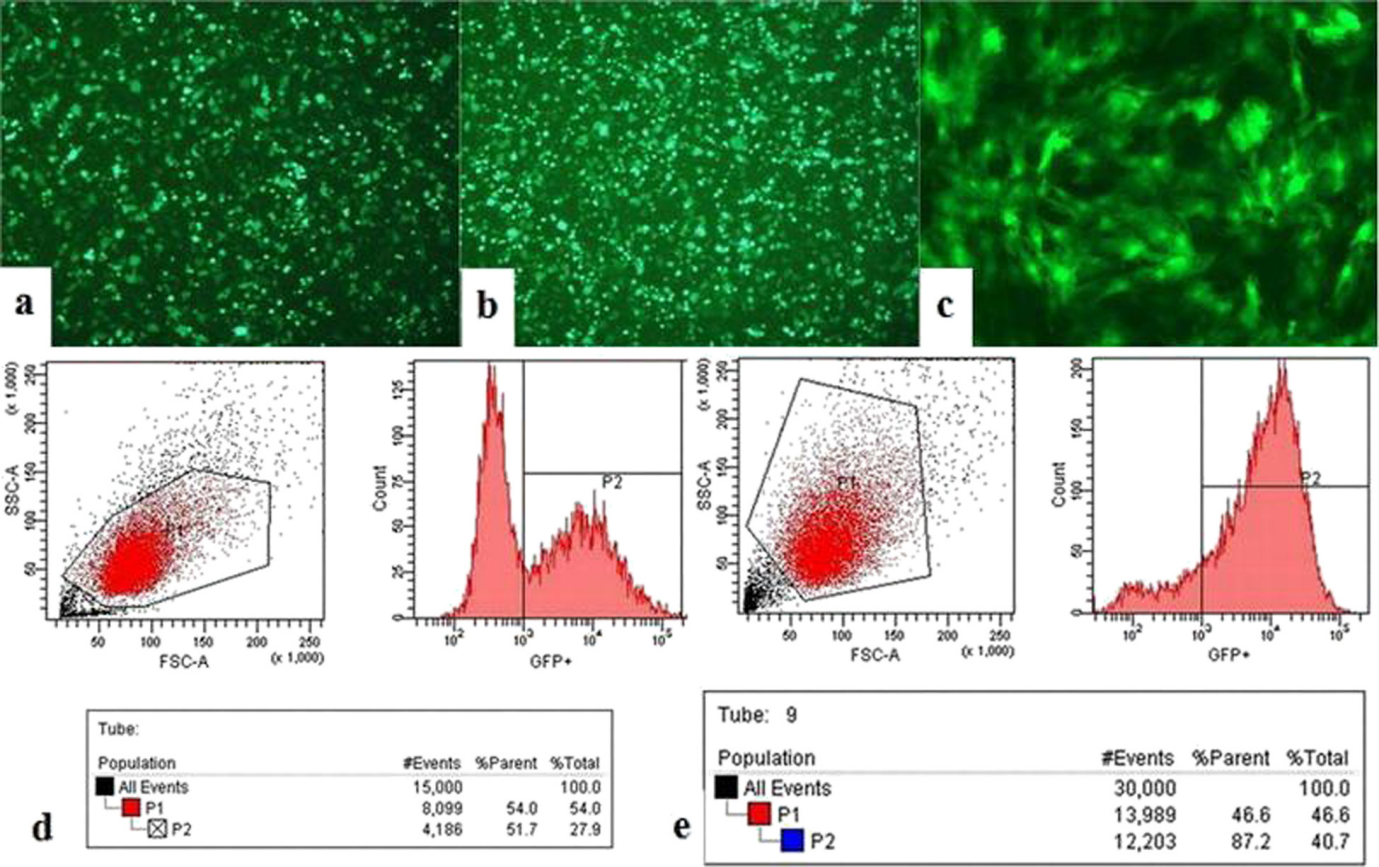
**GFP fluorescence and flow cytometry. (a)** GFP fluorescence imaging showed that 293 T cells were transfected by viral vector after 48 h. **(b)** GFP fluorescence imaging showed that the packaged virus infected 293 T cells after 48 h. **(c)** GFP fluorescence imaging showed that BM-MSCs were infected by the packaged virus. **(d)** The results of flow cytometry on 293 T cells showed that 51.7% of the total number of cells was GFP positive. **(e)** The results of flow cytometry on BM-MSCs showed that 87.2% of the total number of cells was GFP positive.

GFP fluorescence imaging showed that the 293 T cells were transfected by viral vector successfully after 48 h (Figure 1a), and the packaged virus infected 293 T cells successfully after 48 h (Figure 1b). The titer of viral vector was calculated according to the results of flow cytometry (Figure 1d). The title calculation is as follows: titer (293 T-transducing units/ml) = 100,000 (target 293 T cells) × (% of GFP-positive cells/100)/volume of supernatant (ml). The titer of this experiment is 5.17 × 10^8^ TU/ml. BM-MSCs were infected by the packaged virus successfully (Figure 1c). The results of flow cytometry on BM-MSCs (Figure 1e) showed that 87.2% of the total cells were GFP positive.

### Overexpression of SCD1

**Figure 2:**
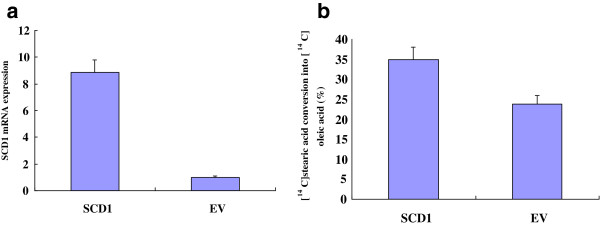
**Realtime PCR and activity results of SCD1.** Realtime PCR and enzyme activity analysis results showed that the amount of SCD1 mRNA **(a)** and enzyme activity **(b)** of the SCD1 overexpressed group was higher than that of the EV group (p < 0.01).

**Figure 3:**
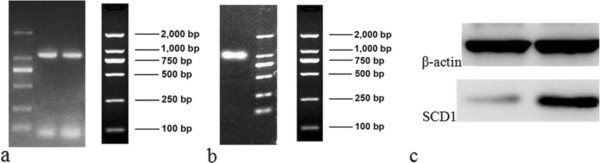
**Vector construction of SCD1 overexpression. (a)** An electrophoresis map of the PCR products of SCD1. **(b)** An electrophoresis map of the PCR products of connected vector pCDH-SCD1. **(c)** Western blot of SCD1 showed that the concentration of SCD1 in the SCD1 overexpressed group is higher than that in the EV group.

RT-PCR showed that the amount of SCD1 mRNA in the SCD1 overexpressed group was 8.86 ± 0.94 and that the amount of the empty vector (EV) group was 1.00 ± 0.09. The expression of SCD1 mRNA of the SCD1 overexpressed group is statistically higher than that of the EV group (p < 0.01) (Figure 2a). Enzyme activity measurement showed that the activity of SCD1 in the SCD1 overexpressed group was 35.87 ± 3.11 and that the activity of the empty vector (EV) group was 23.75 ± 2.16. SCD1 activity of the SCD1 overexpressed group is statistically higher than that of the EV group (p < 0.01) (Figure 2b). A western blot of SCD1 showed that the concentration of SCD1 in the SCD1 overexpressed group was higher than that of the EV group (Figure 3).

### Endothelial cell induction

**Figure 4:**
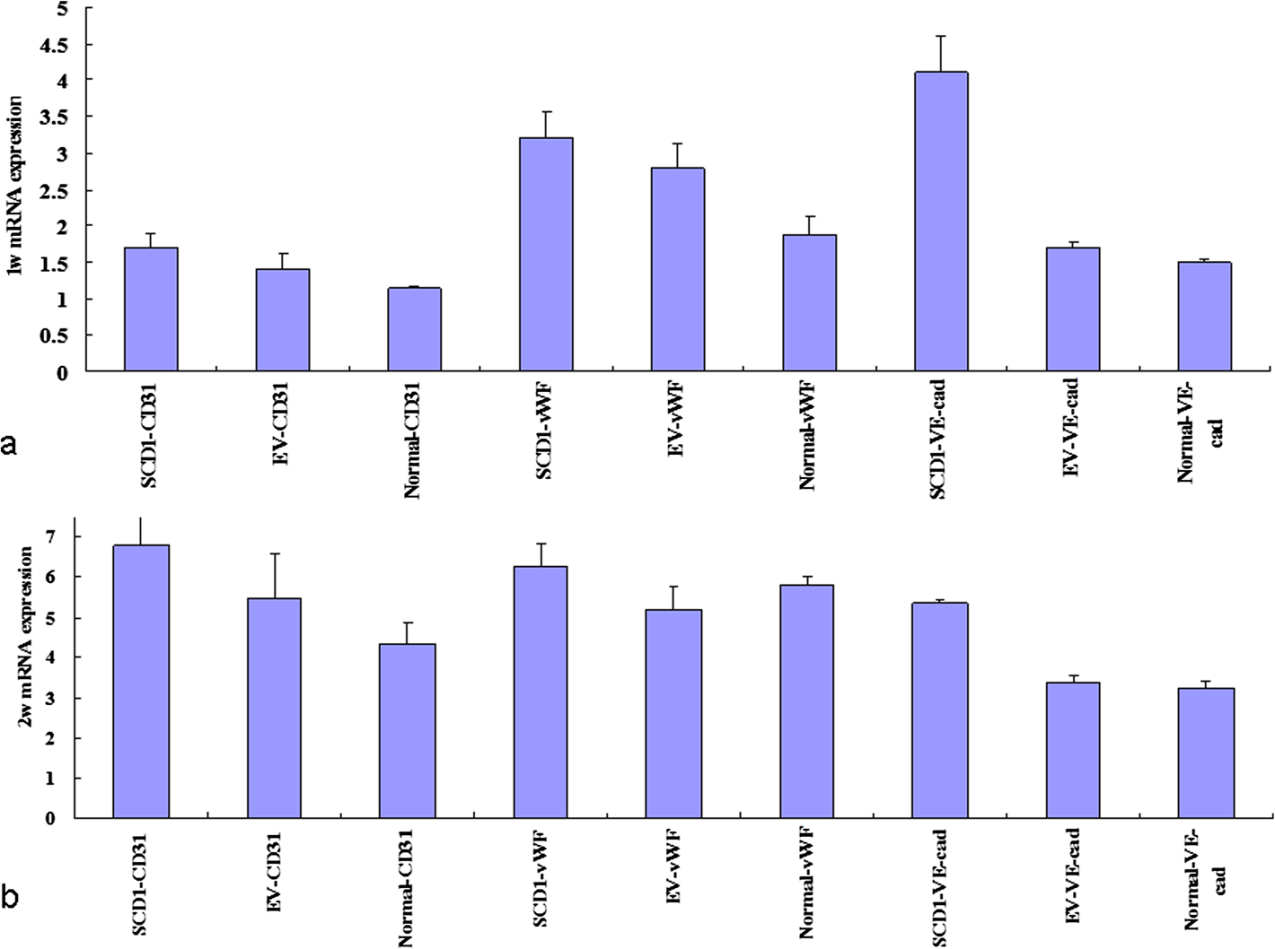
**Realtime PCR results of CD31, vWF and VE-cad.** Realtime PCR results showed that the mRNA amount of CD31, vWF and VE-cad of the SCD1 overexpressed group was higher than that of the EV group and that of the normal group after 1 week **(a)** and 2 weeks **(b)** (p < 0.05).

**Figure 5:**
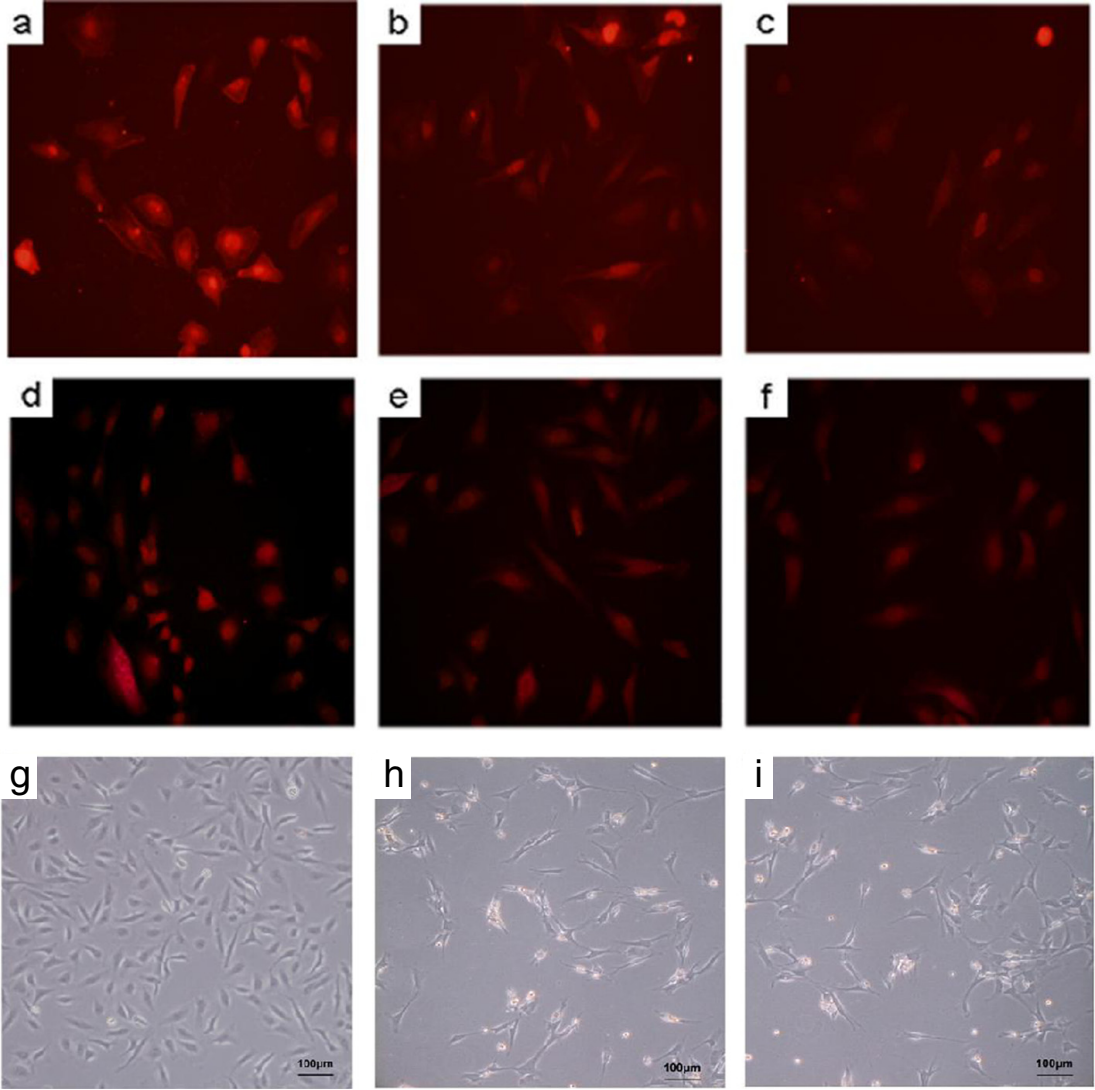
**Immunostaining of CD31 and vWF.** The MSCs was induced into endothelial cells which were also transfected with/without SCD1 gene or empty-vector. Immunocytochemical staining was performed using CD31 and vWF antibodies. Positive staining is visualized by red color for vWF (Figure 5**a, b, c)** or CD31 (Figure 5**d, e, f)**. a/d was referred to SCD1 overexpressed group, b/e was normal group, c/f was empty-vector group. Microscopic image showed that SCD1 overexpressed group **(g)** had more endothelial cells than normal group **(h)** and empty-vector group **(i)**.

The results of fluorescent quantitative PCR (Table 1, Figure 4) showed that the mRNA amount of CD31 of the SCD1 overexpressed group was higher than that of the EV group after 1 week and 2 weeks (p < 0.05), and was significantly higher than that of the normal group after 1 week and 2 weeks (p < 0.01). The mRNA amount of vWF of the SCD1 overexpressed group was higher than that of the EV group after 1 week and 2 weeks (p < 0.05) and that of the normal group after 2 weeks (p < 0.05), and was significantly higher than that of the normal group after 1 week (p < 0.01). The mRNA amount of VE-cad of the SCD1 overexpressed group was significantly higher than that of the EV group and that of the normal group after 1 week and 2 weeks (p < 0.01). Meanwhile, vWF and CD31 staining could be detected and the positive staining is visualized by red color (Figure 5a, b, c for vWF and d, e, f for CD31).

**Table 1:** Results of fluorescent quantitative PCR (mean ± SD)

	**SCD1 (repeating times)**	**EV (repeating times)**	**Normal (repeating times)**	**P value SCD1 vs EV**	**P value SCD1 vs normal**
CD31 (1 week)	1.70 ± 0.18 (6)	1.39 ± 0.23 (6)	1.15 ± 0.02 (6)	0.013*	<0.01**
CD31 (2 weeks)	6.78 ± 0.93 (6)	5.51 ± 1.05 (6)	4.37 ± 0.54 (6)	0.025*	<0.01**
vWF (1 week)	3.21 ± 0.36 (6)	2.80 ± 033 (6)	1.87 ± 0.27 (6)	0.033*	<0.01**
vWF (2 weeks)	6.25 ± 0.56 (6)	5.21 ± 0.57 (6)	5.80 ± 0.20 (6)	0.048*	0.047*
VE-cad (1 week)	4.11 ± 0.51 (6)	1.70 ± 0.07 (6)	1.49 ± 0.05 (6)	<0.01**	<0.01**
VE-cad (2 weeks)	5.36 ± 0.07 (6)	3.37 ± 0.19 (6)	3.24 ± 0.16 (6)	<0.01**	<0.01**

*p value < 0.05 expressed mRNA expression of CD31, vWF or VE-cad of the SCD1 overexpressed group was statistically higher than that of the EV group or that of the normal group after 1 week and 2 weeks.

**p value < 0.01 expressed mRNA expression of CD31, vWF or VE-cad of the SCD1 overexpressed group was statistically significantly higher than that of the EV group or that of the normal group after 1 week and 2 weeks.

## Discussion

SCD1 has been regarded as the switch between fatty acid storage and consumption as well as in promoting or preventing lipid-induced disorders [9,10]. SCD1 is involved in the cellular biosynthesis of MUFAs from SFA substrates. The substrates of SCD1 are mainly stearoyl-CoA (C18:0) and palmitoyl-CoA (C16:0), which are desaturated to oleoyl-CoA (C18:1) and palmitoleoyl-CoA (C16:1), respectively. SCD1 prefers to convert stearate to oleate rather than palmitate to palmitoleate [11], both of which make up the primary storage of unsaturated fatty acids in human adipose tissue [2]. The major MUFAs generated by SCD1 represent the main substrates for synthesis of triglycerides, cholesterol esters and phospholipids. Consistent with its role as a key player in metabolic control, SCD1 activity is tightly regulated, being decreased by unsaturated fatty acids (UFAs) and increased by SFAs [12].

MSCs were found to differentiate into cartilage, bone, fat, muscle, and other connective tissues [6,7] depending on the culture conditions, which include supplementation of lineage-specific inducing agents as well as hormones and growth factors. Studies have demonstrated that mesoderm-derived MSCs originate from precursors with angiogenic potential, called mesenchymomagioblasts, which are identified as MS-CFCs with the potential to differentiate into both MSCs and endothelial cells [13]. In endothelial differentiation system, human bone marrow-derived (CD105+, CD29+, CD34–) MSCs are capable of differentiating into endothelial cells in vitro and acquire major characteristics of mature endothelial-like expression of vWF and CD31 [8]. In addition, MSCs give rise to hematopoiesis-supportive stroma and contribute to the formation of the hematopoietic stem cell niche [14,15] and vascular wall [16].

In this study, GFP fluorescence imaging showed that BM-MSCs were infected by the packaged virus, and RT-PCR results showed that the expression of SCD1 mRNA of the SCD1 overexpressed group is higher than that of the EV group (p < 0.01). So overexpression of SCD1 in BM-MSCs was achieved successfully. Commonly used markers of the endothelium include CD31 and vWF [17]. Vascular endothelial cadherin (VE-cad), a putative member of the type II subfamily [18,19] is specifically expressed in endothelial cells and is not found in any other cell type [20,21]. In order to test the growth of induced endothelial cells, CD31, vWF and VE-cad in MSCs were assayed, and the results of fluorescent quantitative PCR suggested that the mRNA amount of CD31, vWF, VE-cad of the SCD1 overexpressed group was statistically higher than that of the EV group and that of the normal group after 1 week and 2 weeks (p < 0.05). This result indicated that overexpression of SCD1 in BM-MSCs could increases the expression of induced endothelial cells in vitro.

SFAs and lipid oxidation products have been linked with postprandial endothelial dysfunction [4,22] and atherosclerotic disease. Diets rich in SFAs [23] or oxidized fatty acids [24] accelerate the formation of atherosclerotic lesions in animals. High intake of SFAs has been associated with an increased incidence of coronary heart disease (CHD), whereas high intake of MUFAs has been associated with a protective effect [25,26]. Dietary fats can modify CHD risk by their effect on plasma LDL and HDL cholesterol levels [27]. Consumption of SFAs reduces the anti-inflammatory potential of HDL and impairs arterial endothelial function. Consumption of a Mediterranean-type MUFA-diet produces a decrease in plasma levels of vWF, TFPI and PAI-1 plasma levels in young healthy males. Given that these substances are of endothelial origin, it could be suggested that MUFA of the diet has a beneficial effect on endothelial function [28].

A large panel of inflammatory genes are regulated by SFAs. A possible role of SFAs in inflammation has been demonstrated in vitro. When stimulating human cells with palmitic acid, the gene expression and protein production of IL-6 increased [29,30]. The exact mechanisms for these effects are unknown but proposed involved molecules are nuclear factor (NF)-κB and protein kinase C [30]. SFAs represent potential contributors to the vascular inflammation in subjects with metabolic syndrome [31]. In the liver, SCD1 deficiency sensitizes cells to injury [32]. In human myotubes, overexpression of SCD1 enhances triglyceride synthesis and prevents inflammatory and ER-stress responses to palmitate [33]. Overexpression of SCD1 in BM-MSCs might increase the growth of induced endothelial cells by decreasing the amount of SFAs and preventing inflammatory and ER-stress responses.

Study has shown that SCD1 deficiency increases the rate of β-oxidation in soleus and red gastrocnemius muscles by activating of the AMP-activated protein kinase (AMPK) pathway [34,35]. AMPK leads to phosphorylation and inactivation of acetyl-CoA carboxylase resulting in decreased malonyl-CoA content [36]. Malonyl-CoA is both an intermediate in de novo synthesis of fatty acids and an allosteric inhibitor of carnitine palmitoyltransferase 1 (CPT1), the enzyme that transfers long-chain acyl-CoA molecules from the cytosol to the mitochondria where they are oxidized [37]. A decrease in the cellular levels of malonyl-CoA in the liver and skeletal muscles of SCD1−/− mice would thus derepress CPT1, resulting in increased fatty acid oxidation and downregulation of fatty acid synthesis [37]. High expression of SCD1 is corresponded with low rates of fatty acid oxidation (decreased AMPK activity) [38]. Less fat acid oxidation might reduce inflammatory responses and be beneficial for the growth of induced endothelial cells.

SCD1 deficiency results in increased the phosphorylation of the cAMP response element binding protein (CREBP) and the activation of the peroxisome proliferator activated receptor γ (PPARγ) coactivator-1a (PGC-1a) transcription factor through activation of the β3 adrenergic receptor pathway [39], resulting in increased activation of uncoupling protein 1 (UCP1) in BAT of SCD1−/− mice. Increased UCP1 expression uncouples oxidative respiration from ATP synthesis, thereby increasing the rate of basal thermogenesis and consequently, whole body energy expenditure, in SCD1−/− mice [39]. Therefore, high expression of SCD1 might decrease the energy expenditure of cells, helping the growth of induced endothelial cells.

It has been demonstrated that SCD1 modulates the passage of cycling cells through the G1/S boundary and the entry into the apoptotic program, and SCD1 regulates mitogenesis by modulating the rate of fatty acid synthesis, by preventing the toxic accumulation of SFA, and by controlling the supply of MUFA substrates required for lipid biosynthesis and cell proliferation [40]. Overexpression of SCD1 in BM-MSCs might contribute to proliferation of induced endothelial cells by modulating the passage of cycling cells [41].

## Conclusion

This study suggested that overexpression of SCD1 in BM-MSCs could increase the expression of induced endothelial cells in vitro by showing that the mRNA amount of CD31, vWF and VE-cad of BM-MSCs increased after overexpression of SCD1 in vitro. However, the mechanism by which SCD1 increases the expression of induced endothelial cells of BM-MSCs needs further study.

## Methods

### Vector system

The lentiviral vector system (Tronolab) includes pCDH, psPAX2, and pMD. pCDH can express green fluorescent protein (GFP). Both psPAX2 and pMD contain essential elements for virus packaging.

### Vector construction and virus packaging

Using SCD1 gene purchased from OriGene Technologies (SC108809) as a template, fragments of the SCD1 gene were amplified (Figure 3a) using primers capped with BamHI and EcoRI recognition sequences (Table 2 SCD1*). This fragment was then inserted at a unique BamHI site and EcoRI site in the shuttle vector (pCDH) to construct the pCDH-SCD1 plasmid. E. coli was transformed with pCDH-SCD1 and cultured overnight at 37°C. Polymerase chain reaction (PCR) of the connected product was performed (Figure 3b), and the construct was confirmed by DNA sequencing. 293 T cells were transfected by pCDH-SCD1, psPAX2 and pMD. The virus supernatant was collected after 48 h of cultivation. A concentrated solution of virus was made with ultra centrifugation and purification, and the titer of virus was determined. BM-MSCs were then infected by the packaged virus. GFP-positive cells were selected by flow cytometry.

**Table 2:** Primer sequences of SCD1*, β-actin and SCD1**

**Gene**	**Primer sequence**
SCD1*	Forward 5′-CATGGATCCATGCCGGCCCACTTGCTGCAG-3′
	Reverse 5′-TATGAATTCTCAGCCACTCTTGTAGTTTC-3′
β-actin	Forward 5′-CTCCATCCTGGCCTCGCTGT-3′
	Reverse 5′-GCTGTCACCTTCACCGTTCC-3′
SCD1**	Forward 5′-CAGTGTGTTCGTTGCCACTT-3′-
	Reverse 5′--GGTAGTTGTGGAAGCCCTCA-3′-

*Primer used to draw SCD1 fragments for pCDH-SCD1 construction from SCD1 gene.

**Primer used to monitor the expression of SCD1 in BM-MSCs.

### Western blot

Cells were lysed in RIPA buffer, and protein extraction was carried out on ice. Protein concentration was determined by BCA assay. Eight μl of total protein lysate were mixed with SDS loading buffer, and proteins were separated by electrophoresis on 12% SDS-PAGE. After that, proteins were transferred onto PVDF membranes. After blocking with 5% skim milk in PBS for 2 hours, membranes were washed and incubated sequentially with primary anti-SCD1 antibody (ab 19862; Abcam, Cambridge, USA) and anti-mouse secondary antibody-horseradish conjugate (Abcam). Western blots were developed colorimetrically (Figure 3c).

### Overexpression of SCD1

Total RNA was extracted from BM-MSCs using Trizol (Invitrogen). Prior to real time PCR (RT-PCR), the RNA was treated with DNase I. The purified RNA was used for first-strand cDNA synthesis, and reverse transcription was performed using M-MLV reverse transcriptase with oligo-dT primers according to the manufacturer’s instructions (Promega). The 170-bp fragment of the SCD1 gene was amplified using primers (Table 2 SCD1**), with β-actin (Table 2) used as an internal control gene.

### Determination of Scd1 activity

We evaluated Scd1 activity by measuring the conversion of [^14^C] stearic acid into [^14^C] oleic acid after overexpression SCD1. Cells were incubated with 3 μM (0.25 μCi/dish of [^14^C] stearic acid) for 6 h at 37°C in 5% CO_2_ incubator. Cells were collected and total lipids were extracted according to Bligh and Dyer method [42]. Lipids were saponified and esterified. Radiolabelled fatty acid methyl esters were separated by RP-HPLC and detected on line by a radioisotope detector (Packard Flow Scintillation Analyser, PerkinElmer Life Sciences Inc., Wellesley, MA). The [^14^C] oleic acid/([^14^C] oleic and stearic acids) ratio was determined as Scd activity [43,44].

### Endothelial cell induction

BM-MSCs were cultured in Dulbecco’s modified Eagle’s medium (DMEM) containing 10% fetal bovine serum (FBS) at 37°C in a humidified atmosphere of 5% CO and 95% air, according to the standard procedure. The cell lines were routinely passaged using an enzymatic solution consisting of 0.25% (w/v) trypsin for 5 min at 37°C. The third generation cells, with 1 × 10^4^ cells/well, were transferred onto a 12-well plate. After 24 h, endothelial cell induction was performed with induction medium (low glucose DMEM containing 10% FBS). Fluorescent quantitative PCR of CD31, vWF and VE-cad was performed after 1 week and 2 weeks to test the growth of induced endothelial cells.

### Immunostaining

A total of 1×10^5^ cells were incubated at 4°C with a mouse mono-clonal anti-CD31 antibody (Southern Biotechnology, AL, USA). After 45 min, the cells were washed with PBS and stained with an Alexa 488-conjugated goat anti-mouse anti-body (Invitrogen) for 30 min at 4°C in the dark. The cells were also rehydrated and incubated for 2 h with anti-von Willebrand factor (vWF) (DakoCytomation, clone F8/86). Subsequently, rabbit-anti-mouse immunoglobulin (DakoCytomation) was added for 45 min. Sections and cells were photographed using a Sony color video camera.

### Fluorescent quantitative PCR

Total RNA extraction was performed according to the instruction of RNA extraction kit (QIAGEN). RNA reverse transcription was performed following the instruction of M-MLV (Promega). Fluorescent quantitative PCR was then performed to detect the expression of CD31, vWF and VE-cad, with β-actin used as an internal control gene. Primer sequences were as follows (Table 3). The amount of PCR copies was obtained according to the standard curve.

**Table 3:** Primer sequences of CD31, vWF and VE-cad

**Gene**	**Primer sequence**
β-actin	Forward 5′-ATCGTGGGCCGCCCTAGGCA-3′
	Reverse 5′-TGGCCTTAGGGTTCAGAGGGG-3′
vWF	Forward 5′-CCCACCGGATGGCTAGGTATT-3′
	Reverse 5′-GAGGCGGATCTGTTTGAGGTT-3′
CD31	Forward 5′-GGACTGGCCCTGTCACGTT-3′
	Reverse 5′-TTGTTCATGGTGCCAAAACACT-3′
VE-cad	Forward 5′-GGCCAACGAATTGGATTCTA-3′
	Reverse 5′-GTTTACTGGCACCACGTCCT-3′

### Statistical analysis

All experiments were repeated six times. All values were presented as the means ± SD. Statistically significant differences between groups were assessed by an unpaired Student’s t-test. Differences of p <0.05 were considered to be statistically significant.

## Competing interests

The authors declare that they have no competing interests.

## Authors’ contributions

YL (Yuanshan Lu) and JT designed the experiment and protocols and wrote the manuscript. YL (Yuanshan Lu) performed Vector construction and virus package, BM-MSCs infection, flow cytometry analysis. ZZ participated in cell cultivating. BD performed real-time PCR, western blot. MG performed Immunostaining. YL performed statistical analysis. YL (Yuanshan Lu) and JT interpreted the data. All authors read and approved the final manuscript.

## References

[B1] Man WC Miyazaki M Chu K Ntambi JM Membrane topology of mouse stearoyl-CoA desaturase 1 J Biol Chem 2006 281 2 1251 1260 10.1074/jbc.M508733200 16275639

[B2] Insull W Jr Bartsch GE Fatty acid composition of human adipose tissue related to age, sex, and race Am J Clin Nutr 1967 20 1 13 23 6017005 10.1093/ajcn/20.1.13

[B3] Sarabi M Vessby B Millgard J Lind L Endothelium-dependent vasodilation is related to the fatty acid composition of serum lipids in healthy subjects Atherosclerosis 2001 156 2 349 355 10.1016/S0021-9150(00)00658-4 11395031

[B4] Nicholls SJ Lundman P Harmer JA Cutri B Griffiths KA Rye KA Barter PJ Celermajer DS Consumption of saturated fat impairs the anti-inflammatory properties of high-density lipoproteins and endothelial function J Am Coll Cardiol 2006 48 4 715 720 10.1016/j.jacc.2006.04.080 16904539

[B5] Chen Y Osika W Dangardt F Gan LM Strandvik B Friberg P High levels of soluble intercellular adhesion molecule-1, insulin resistance and saturated fatty acids are associated with endothelial dysfunction in healthy adolescents Atherosclerosis 2010 211 2 638 642 10.1016/j.atherosclerosis.2010.03.013 20362293

[B6] Tontonoz P Spiegelman BM Fat and beyond: the diverse biology of PPARgamma Annu Rev Biochem 2008 77 289 312 10.1146/annurev.biochem.77.061307.091829 18518822

[B7] Matsuzawa Y Adipocytokines and metabolic syndrome Semin Vasc Med 2005 5 1 34 39 10.1055/s-2005-871744 15968578

[B8] Tao J Sun Y Wang QG Liu CW Induced endothelial cells enhance osteogenesis and vascularization of mesenchymal stem cells Cells Tissues Organs 2009 190 4 185 193 10.1159/000218139 19420896

[B9] Dobrzyn P Jazurek M Dobrzyn A Stearoyl-CoA desaturase and insulin signaling–what is the molecular switch? Biochim Biophys Acta 2010 1797 6–7 1189 1194 20153289 10.1016/j.bbabio.2010.02.007

[B10] Sampath H Miyazaki M Dobrzyn A Ntambi JM Stearoyl-CoA desaturase-1 mediates the pro-lipogenic effects of dietary saturated fat J Biol Chem 2007 282 4 2483 2493 10.1074/jbc.M610158200 17127673

[B11] Ntambi JM Miyazaki M Regulation of stearoyl-CoA desaturases and role in metabolism Prog Lipid Res 2004 43 2 91 104 10.1016/S0163-7827(03)00039-0 14654089

[B12] Liew CF Groves CJ Wiltshire S Zeggini E Frayling TM Owen KR Walker M Hitman GA Levy JC O’Rahilly S Hattersley AT Johnston DG McCarthy MI Analysis of the contribution to type 2 diabetes susceptibility of sequence variation in the gene encoding stearoyl-CoA desaturase, a key regulator of lipid and carbohydrate metabolism Diabetologia 2004 47 12 2168 2175 10.1007/s00125-004-1575-4 15662557

[B13] Vodyanik MA Yu J Zhang X Tian S Stewart R Thomson JA Slukvin II A mesoderm-derived precursor for mesenchymal stem and endothelial cells Cell Stem Cell 2010 7 6 718 729 10.1016/j.stem.2010.11.011 21112566 PMC3033587

[B14] Blazsek I Chagraoui J Peault B Ontogenic emergence of the hematon, a morphogenetic stromal unit that supports multipotential hematopoietic progenitors in mouse bone marrow Blood 2000 96 12 3763 3771 11090058

[B15] Muguruma Y Yahata T Miyatake H Sato T Uno T Itoh J Kato S Ito M Hotta T Ando K Reconstitution of the functional human hematopoietic microenvironment derived from human mesenchymal stem cells in the murine bone marrow compartment Blood 2006 107 5 1878 1887 10.1182/blood-2005-06-2211 16282345

[B16] Crisan M Yap S Casteilla L Chen CW Corselli M Park TS Andriolo G Sun B Zheng B Zhang L Norotte C Teng PN Traas J Schugar R Deasy BM Badylak S Buhring HJ Giacobino JP Lazzari L Huard J Peault B A perivascular origin for mesenchymal stem cells in multiple human organs Cell Stem Cell 2008 3 3 301 313 10.1016/j.stem.2008.07.003 18786417

[B17] Hormia M Lehto VP Virtanen I Identification of UEA I-binding surface glycoproteins of cultured human endothelial cells Cell Biol Int Rep 1983 7 6 467 475 10.1016/0309-1651(83)90136-4 6883521

[B18] Nollet F Kools P van Roy F Phylogenetic analysis of the cadherin superfamily allows identification of six major subfamilies besides several solitary members J Mol Biol 2000 299 3 551 572 10.1006/jmbi.2000.3777 10835267

[B19] Shimoyama Y Tsujimoto G Kitajima M Natori M Identification of three human type-II classic cadherins and frequent heterophilic interactions between different subclasses of type-II classic cadherins Biochem J 2000 349 Pt 1 159 167 10861224 10.1042/0264-6021:3490159 PMC1221133

[B20] Lampugnani MG Resnati M Raiteri M Pigott R Pisacane A Houen G Ruco LP Dejana E A novel endothelial-specific membrane protein is a marker of cell-cell contacts J Cell Biol 1992 118 6 1511 1522 10.1083/jcb.118.6.1511 1522121 PMC2289607

[B21] Breier G Breviario F Caveda L Berthier R Schnurch H Gotsch U Vestweber D Risau W Dejana E Molecular cloning and expression of murine vascular endothelial-cadherin in early stage development of cardiovascular system Blood 1996 87 2 630 641 8555485

[B22] Williams MJ Sutherland WH McCormick MP de Jong SA Walker RJ Wilkins GT Impaired endothelial function following a meal rich in used cooking fat J Am Coll Cardiol 1999 33 4 1050 1055 10.1016/S0735-1097(98)00681-0 10091835

[B23] Rudel LL Parks JS Sawyer JK Compared with dietary monounsaturated and saturated fat, polyunsaturated fat protects African green monkeys from coronary artery atherosclerosis Arterioscler Thromb Vasc Biol 1995 15 12 2101 2110 10.1161/01.ATV.15.12.2101 7489230

[B24] Staprans I Rapp JH Pan XM Hardman DA Feingold KR Oxidized lipids in the diet accelerate the development of fatty streaks in cholesterol-fed rabbits Arterioscler Thromb Vasc Biol 1996 16 4 533 538 10.1161/01.ATV.16.4.533 8624775

[B25] Nordoy A Goodnight SH Dietary lipid and thrombosis: relationship to atherosclerosis Arterioscler 1990 10 2 149 163 10.1161/01.ATV.10.2.149 2180393

[B26] Shekelle RB Shryock AM Paul O Lepper M Stamler J Liu S Raynor WJ Jr Diet, serum cholesterol, and death from coronary heart disease: the western electric study N Engl J Med 1981 304 2 65 70 10.1056/NEJM198101083040201 7442730

[B27] Mensink RP Katan MB Effect of dietary fatty acids on serum lipids and lipoproteins: a meta-analysis of 27 trials Arterioscler Thromb 1992 12 8 911 919 10.1161/01.ATV.12.8.911 1386252

[B28] Perez-Jimenez F Castro P Lopez-Miranda J Paz-Rojas E Blanco A Lopez-Segura F Velasco F Marin C Fuentes F Ordovas JM Circulating levels of endothelial function are modulated by dietary monounsaturated fat Atherosclerosis 1999 145 2 351 358 10.1016/S0021-9150(99)00116-1 10488963

[B29] Weigert C Brodbeck K Staiger H Kausch C Machicao F Haring HU Schleicher ED Palmitate, but not unsaturated fatty acids, induces the expression of interleukin-6 in human myotubes through proteasome-dependent activation of nuclear factor-kappaB J Biol Chem 2004 279 23 23942 23952 10.1074/jbc.M312692200 15028733

[B30] Ajuwon KM Spurlock ME Palmitate activates the NF-kappaB transcription factor and induces IL-6 and TNFalpha expression in 3 T3-L1 adipocytes J Nutr 2005 135 8 1841 1846 16046706 10.1093/jn/135.8.1841

[B31] Krogmann A Staiger K Haas C Gommer N Peter A Heni M Machicao F Haring HU Staiger H Inflammatory response of human coronary artery endothelial cells to saturated long-chain fatty acids Microvasc Res 2011 81 1 52 59 10.1016/j.mvr.2010.11.008 21112343

[B32] Nichols LA Jackson DE Manthey JA Shukla SD Holland LJ Citrus flavonoids repress the mRNA for stearoyl-CoA desaturase, a key enzyme in lipid synthesis and obesity control, in rat primary hepatocytes Lipids Health Dis 2011 10 1476 511 10.1186/1476-511X-10-36 PMC3056818 21345233

[B33] Peter A Weigert C Staiger H Machicao F Schick F Machann J Stefan N Thamer C Haring HU Schleicher E Individual stearoyl-coa desaturase 1 expression modulates endoplasmic reticulum stress and inflammation in human myotubes and is associated with skeletal muscle lipid storage and insulin sensitivity in vivo Diabetes 2009 58 8 1757 1765 10.2337/db09-0188 19478146 PMC2712792

[B34] Dobrzyn P Dobrzyn A Miyazaki M Cohen P Asilmaz E Hardie DG Friedman JM Ntambi JM Stearoyl-CoA desaturase 1 deficiency increases fatty acid oxidation by activating AMP-activated protein kinase in liver Proc Natl Acad Sci U S A 2004 101 17 6409 6414 10.1073/pnas.0401627101 15096593 PMC404058

[B35] Dobrzyn A Dobrzyn P Lee SH Miyazaki M Cohen P Asilmaz E Hardie DG Friedman JM Ntambi JM Stearoyl-CoA desaturase-1 deficiency reduces ceramide synthesis by downregulating serine palmitoyltransferase and increasing beta-oxidation in skeletal muscle Am J Physiol Endocrinol Metab 2005 288 3 E599 607 15562249 10.1152/ajpendo.00439.2004

[B36] Hardie DG Pan DA Regulation of fatty acid synthesis and oxidation by the AMP-activated protein kinase Biochem Soc Trans 2002 30 Pt 6 1064 1070 12440973 10.1042/bst0301064

[B37] McGarry JD Mannaerts GP Foster DW A possible role for malonyl-CoA in the regulation of hepatic fatty acid oxidation and ketogenesis J Clin Invest 1977 60 1 265 270 10.1172/JCI108764 874089 PMC372365

[B38] Dobrzyn A Dobrzyn P Stearoyl-CoA desaturase–a new player in skeletal muscle metabolism regulation J Physiol Pharmacol 2006 57 Suppl 10 31 42 17242489

[B39] Lee SH Dobrzyn A Dobrzyn P Rahman SM Miyazaki M Ntambi JM Lack of stearoyl-CoA desaturase 1 upregulates basal thermogenesis but causes hypothermia in a cold environment J Lipid Res 2004 45 9 1674 1682 10.1194/jlr.M400039-JLR200 15210843

[B40] Hess D Chisholm JW Igal RA Inhibition of stearoylCoA desaturase activity blocks cell cycle progression and induces programmed cell death in lung cancer cells PLoS One 2010 5 6 e11394 10.1371/journal.pone.0011394 20613975 PMC2894866

[B41] Tao J Shi J Lu Y Dou B Zhou Z Gao M Zhu Z Overexpression of stearoyl-CoA desaturase 1 in bone-marrow mesenchymal stem cells increases osteogenesis Panminerva Med 2013 55 3 283 289 24088802

[B42] Bligh EG Dyer WJ A rapid method for total lipid extraction and purification Can J Biochem Physiol 1959 37 911 917 10.1139/o59-099 13671378

[B43] Minville-Walz M Pierre AS Pichon L Bellenger S Fevre C Bellenger J Tessier C Narce M Rialland M Inhibition of stearoyl-coa desaturase 1 expression induces chop-dependent cell death in human cancer cells PloS one 2010 5 e14363 10.1371/journal.pone.0014363 21179554 PMC3002938

[B44] Scaglia N Caviglia JM Igal RA High stearoyl-coa desaturase protein and activity levels in simian virus 40 transformed-human lung fibroblasts Biochimica et biophysica acta 2005 1687 141 151 10.1016/j.bbalip.2004.11.015 15708362

